# The Biosynthesis and Metabolism of the *N*-Acylated Aromatic Amino Acids: *N*-Acylphenylalanine, *N*-Acyltyrosine, *N*-Acyltryptophan, and *N*-Acylhistidine

**DOI:** 10.3389/fmolb.2021.801749

**Published:** 2022-01-03

**Authors:** Suzeeta Bhandari, Kirpal S. Bisht, David J. Merkler

**Affiliations:** Department of Chemistry, University of South Florida, Tampa, FL, United States

**Keywords:** aromatic amino acids, lipo-amino acids, fatty acid amides, biosynthesis, degradation, metabolism

## Abstract

The fatty acid amides are a family of lipids composed of two chemical moieties, a fatty acid and a biogenic amine linked together in an amide bond. This lipid family is structurally related to the endocannabinoid anandamide (*N*-arachidonoylethanolamine) and, thus, is frequently referred to as a family of endocannabinoid-related lipids. The fatty acid amide family is divided into different classes based on the conjugate amine; anandamide being a member of the *N*-acylethanolamine class (NAE). Another class within the fatty acid amide family is the *N*-acyl amino acids (NA-AAs). The focus of this review is a sub-class of the NA-AAs, the *N*-acyl aromatic amino acids (NA-ArAAs). The NA-ArAAs are not broadly recognized, even by those interested in the endocannabinoids and endocannabinoid-related lipids. Herein, the NA-ArAAs that have been identified from a biological source will be highlighted and pathways for their biosynthesis, degradation, enzymatic modification, and transport will be presented. Also, information about the cellular functions of the NA-ArAAs will be placed in context with the data regarding the identification and metabolism of these *N*-acylated amino acids. A review of the current state-of-knowledge about the NA-ArAAs is to stimulate future research about this underappreciated sub-class of the fatty acid amide family.

## Introduction

The fatty acid amides are a biologically important family of lipids resulting from a fatty acid and a biogenic amine linked together in an amide bond (Ni et al., 2021). Anandamide (*N*-arachidonoylethanolamine) is the most studied member of this lipid family. The *N*-acyl amino acids are members of the fatty acid amide family with an amino acid as the biogenic amine. The occurrence of *N*-acylated amino acids in biological systems has long been known. A few early examples include urinary *N*-isovaleroylglycine from patients suffering from isovaleric acidemia (Tanaka and Isselbacher, 1967) and *N*-acetylglutamate as an allosteric activator of carbamoyl phosphate synthetase I (Grisolia and Cohen, 1953). Knowledge of the *N*-acylated amino acids was generally limited to short-chain NA-AAs because the *N*-acetylated derivatives of all the amino acids have been identified (Wishart et al., 2018) and specific short-chain NA-AAs are biomarkers for different metabolic diseases (Goldstein, 1963; Millington et al., 1991; Loots et al., 2005; Scolamiero et al., 2015). There were a few reports of NA-AAs with acyl chains of six carbon atoms or longer (often called the lipo-amino acids) from living organisms (Fukui and Axelrod, 1961; Hunter and James, 1963; Garg and Murti, 1970; Baretz et al., 1976; Pitt, 1993), but such molecules were not widely understood or appreciated.

Interest in the lipo-amino acids has increased dramatically over the last ∼30 years driven by discoveries showing the biological importance of these molecules and of other structurally related lipo-amides, including the myristoylation of the N-terminal glycine of eukaroytic proteins (Wang et al., 2021), *N*-acylhomoserine lactones as quorum sensing molecules in bacteria (Càmara et al., 1998), *N*-(17-hydroxylinolenoyl)-L-glutamine (volicitin) as an elicitor of plant volatiles from caterpillars (Alborn et al., 1997), anandamide as the endogenous ligand of the CB_1_ receptor (Devane et al., 1992), and oleamide as a regulator of the sleep/wake cycle (Cravatt et al., 1995). Anandamide is a member of the endocannabinoid family of cell signaling lipids. The long-chain NA-AAs are structurally related to anandamide and, thus, are often referred to as endocannabinoid-related lipids.

**FIGURE 1:**
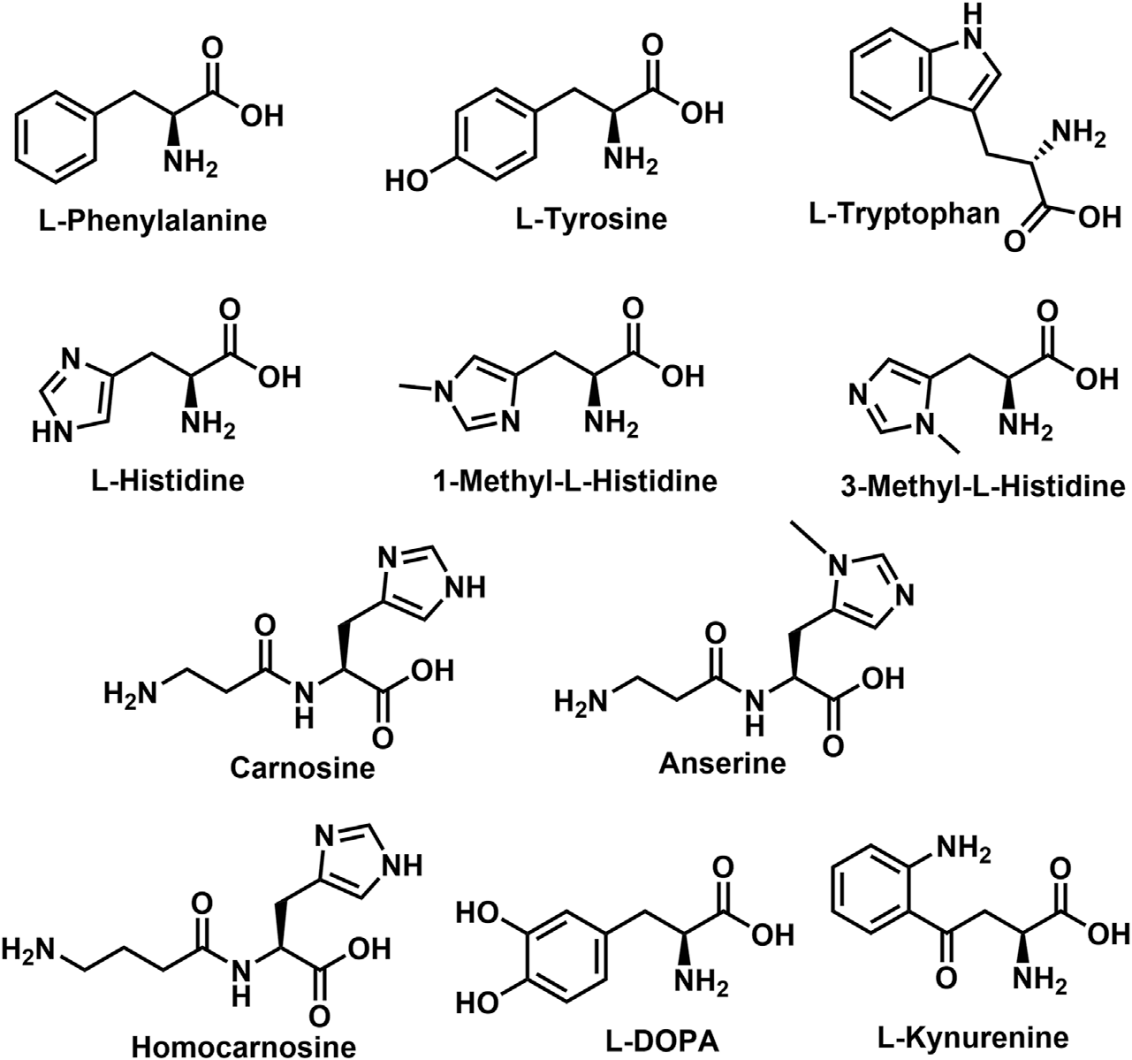
Structures of the Aromatic Amino Acids and Aromatic Amino Acid related biomolecules.

A sub-class of the NA-AAs, the *N*-acyl aromatic amino acids (NA-ArAAs) are not broadly recognized, even by those interested in the endocannabinoid-related lipids, but several of the NA-ArAAs have been identified from a biological source. This review is focused on these topics: identification of the NA-ArAAs from living systems and pathways for their biosynthesis, metabolism, and degradation. Included in the review is information about NA-ArAAs related to the “common” aromatic amino acids, 1-methylhistidine, 3-methylhistidine, anserine, carnosine, homocarnosine, l-DOPA (3,4-dihydroxy-L-phenylalanine), and kynurenine (Figure 1). Little regarding the biological function of the NA-ArAAs is included herein because knowledge of their cellular roles is limited and their cellular roles could differ from organism-to-organism. Other reviews of the lipo-amino acids have discussed the possible functions of these molecules in the cell (Bradshaw et al., 2009a; Hanuš et al., 2014; Anderson and Merkler, 2017; Burstein, 2018; Battista et al., 2019; Prakash and Kamlekar, 2021). This is the first review solely dedicated to the *N*-acylated aromatic amino acids.

## Biological Occurrence

One concern in reviewing the NA-ArAA field was nomenclature: would important publications be missed because members of the *N*-acyl amino acid family are identified by a name that does not correlate well to their structure. For example, most of the published work on *N*-arachidonoylethanolamine is found using “anandamide” as the search term. To circumvent this problem, we systematically built the structures for each of the NA-ArAAs in ChemDraw, starting with the *N*-acetyl derivative, and then used the search function in ChemDraw to identify publications related to the exact structure in SciFinder. Publications identified by SciFinder fostered additional searches in PubMed, Google Scholar, Century of Science, and the Human Metabolite database. The NA-ArAAs that have been identified and characterized from a living system are shown in Table 1. There is nothing in Table 1 about 1-methylhistidine, 3-methylhistidine, anserine, carnosine, homocarnosine, l-DOPA, and kynurenine because the only known acylated derivative of these amino acids is the *N*-acetyl version. All have been identified in human (Wishart et al., 2018) and other mammals (Sasaoka et al., 1982; O’Dowd et al., 1988; O’Dowd et al., 1992) except for *N*-acetyl-l-DOPA, which has only been identified in *Streptomyces* (Solecka et al., 2012). We have omitted some information about the NA-ArAAs from Table 1 to keep this table manageable. The omitted data concerned the biological source of the NA-ArAAs and their cellular concentrations. Many of the NA-ArAAs have been identified from more than one organism. One example is *N*-palmitoyl-phenylalanine, which has been identified from bamboo (Ren et al., 2019), *Drosophila melanogaster* (Tortoriello et al., 2013), soil microorganisms (Clardy and Brady, 2007), rat brain (Tan et al., 2010), and mouse (plasma and eight different regions of the brain) (Leishman et al., 2016). Concentration data for many of the NA-ArAAs listed in Table 1 is not available. For such NA-ArAAs, the lipo-amino acid was detected and characterized, but not quantified. For other NA-ArAAs, only a concentration ratio is reported because a significant concentration change for that NA-ArAA could serve as a disease biomarker (Obianyo et al., 2015; Qi et al., 2017; Bonte et al., 2019; Shoaib et al., 2020). For the NA-ArAAs that have been quantified, concentrations are reported in a diversity of units, including molarity, mass/volume, mass per gram of tissue, mass per gram of wet weight of tissue, or mass per mass of creatine. Another complicating factor is how the concentration data was generated and/or reported. For example, the concentration of *N*-acetyl-3-methylhistidine in rat was reported as a production rate, 2.04 ± 0.13 µmole/24 h/rat (Sasaoka et al., 1982), while those for *N*-acetylated derivatives of histidine, 1-methylhistidine, carnosine, and homocarnosine were from rabbit heart perfusates (O’Dowd et al., 1992). Lastly, the concentration data for a few of the NA-ArAAs have been measured from different organisms or from different tissues of the same organism. For example, the concentrations of *N*-arachidonoyl, *N*-docosahexaenoyl-, *N*-linoleoyl-, *N*-oleoyl-, *N*-palmitoyl-, and *N*-stearoyl-derivatives of Trp, Tyr, and Phe from eight different mouse brain regions were published (Leishman et al., 2016). Readers interested in either the biological source and/or the concentrations for a NA-ArAA listed in Table 1 can obtain this information from the cited references or from a search in one of the databases mentioned above.

**TABLE 1:** *N*-Acyl Aromatic Amino Acids Identified from Living Systems.

l-Amino Acids^a^					
*N*-Acyl Group	Phe	Tyr	Trp	His	References
Acetyl [CH_3_-CO	D	D,Q	D	D,Q	O’Dowd et al. (1988)
					O’Dowd et al. (1992)
					Togashi et al. (1998)
					Tanaka et al. (2015)
					Yu et al. (2014)
					Wishart et al. (2018)
Propionyl [CH_3_-CH_2_-CO-]	NF	NF	NF	NF	
Lactoyl [CH_3_-CH(OH)-CO-]	D	D	NF	NF	Pietrowska et al. (2018)
					Bonte et al. (2019)
					Qian et al. (2021)
Malonyl [HOOC-CH_2_-CO-]	D	D	D	NF	Feldberg et al. (2018)
					Obianyo et al. (2015)
					Farag and Shakour, (2019)
Butyryl [CH_3_-CH_2_-CH_2_-CO-]	NF	D,Q	NF	NF	Hammerl et al. (2017)
Succinoyl [HOOC-CH_2_-CH_2_-CO-]	NF	NF	D,Q	NF	Yu et al. (2014)
*α*-Malyl [HOOC-CH(OH)-CH_2_-CO-]	NF	D	D,Q	NF	Yu et al. (2014)
					Feldberg et al. (2018)
*β*-Malyl [HOOC-CH_2_-CH-(OH)-CO-]	NF	NF	D,Q	NF	Yu et al. (2014)
Crotonyl [CH_3_-CH = CH-CO-, *trans*]	NF	NF	NF	NF	
Isocrotonyl [CH_3_-CH = CH-CO-, *cis*]	NF	NF	NF	NF	
Isobutyryl [(CH_3_)_2_-CH-CO-]	NF	NF	NF	NF	
Pentanoyl (Valeroyl) [CH_3_-(CH_2_)_3_-CO-]	NF	NF	NF	NF	
Isovaleroyl [(CH_3_)_2_-CH-CH_2_-CO-]	D	D	D	D	Loots et al. (2005)
Pivaloyl [(CH_3_)_3_-C-CO-]	NF	NF	NF	NF	
Hexanoyl (Caproyl) [CH_3_-(CH_2_)_4_-CO-]	NF	D,Q	NF	D	Hammerl et al. (2017)
					Tørring et al. (2017)
Heptanoyl [CH_3_-(CH_2_)_5_-CO-]	NF	NF	NF	NF	
Octanoyl (Capryloyl) [CH_3_-(CH_2_)_6_-CO-]	NF	D,Q	D	D	Brady and Clardy (2000)
					Brady et al. (2004)
					Hammerl et al. (2017)
					Tørring et al. (2017)
Nonanoyl (Pelargonoyl) [CH_3_-(CH_2_)_7_-CO-]	NF	D	NF	NF	Brady and Clardy (2000)
					Brady et al. (2004)
Decanoyl [CH_3_-(CH_2_)_8_-CO-]	NF	D	NF	D	Brady and Clardy (2000)
					Brady et al. (2004)
					Tørring et al. (2017)
Decenoyl [CH_3_-(CH_2_)_6_-CH = CH-CO-, 2-*cis*]	NF	NF	NF	D	Brady et al. (2004)
[CH_3_-(CH_2_)_6_-CH = CH-CO-, 2-*trans*]	NF	NF	NF	D	Tørring et al. (2017)
[CH_3_-(CH_2_)_5_-CH = CH-CH_2_-CO-, 3-*cis*]	NF	NF	NF	D	
(2-Hexylcyclopropyl)acetyl [*cis*-cascarilloyl) [CH_3_-(CH_2_)_5_-cyclopropyl-CH_2_-CO-]	NF	NF	NF	D	Tørring et al. (2017)
Undecanoyl [CH_3_-(CH_2_)_9_-CO-]	NF	D	NF	NF	Brady and Clardy (2000)
					Brady et al. (2004)
					Lee et al. (2019)
Lauroyl [CH_3_-(CH_2_)_10_-CO-]	D	D	NF	NF	Brady and Clardy (2000)
					Brady et al. (2004)
					Yeo et al. (2007)
					Craig and Brady (2011)
					Lee et al. (2019)
Tridecanoyl [CH_3_-(CH_2_)_11_-CO-]	D	D	NF	NF	Brady and Clardy (2000)
					Brady et al. (2004)
					Lee et al. (2019)
11-Methyldodecanoyl [(CH_3_)_2_-CH-(CH_2_)_9_-CO-]	D	NF	NF	NF	Craig and Brady (2011)
Myristoyl [CH_3_-(CH_2_)_12_-CO-]	D,Q	D	D	NF	Brady et al. (2004)
					Clardy and Brady (2007)
					Long et al. (2016)
					Thies et al. (2016)
					Lee et al. (2019)
Myristoleoyl [CH_3_-(CH_2_)_3_-CH = CH-(CH_2_)_7_-CO-, *cis*]	NF	NF	NF	NF	
7-Tetradecenoyl [CH_3_-(CH_2_)_5_-CH = CH-(CH_2_)_5_-CO-, *cis*]	NF	D	NF	NF	Brady and Clardy (2000)
Pentadecanoyl [CH_3_-(CH_2_)_13_-CO-]	D	D	NF	NF	Brady and Clardy (2000)
					Brady et al. (2004)
					Clardy and Brady (2007)
					Lee et al. (2019)
8-Pentadecenoyl [CH_3_-(CH_2_)_5_-CH = CH-(CH_2_)_6_-CO-, *cis*]	NF	D	NF	NF	Brady and Clardy (2000)
Palmitoyl [CH_3_-(CH_2_)_14_-CO-]	D,Q	D,Q	D,Q	D	Brady and Clardy (2000)
					Brady et al. (2004)
					Brady and Clardy (2005)
					Clardy and Brady (2007)
					Tan et al. (2010)
					Tortoriello et al. (2013)
					Leishman et al. (2016)
					Long et al. (2016)
					Ren et al. (2019)
					Berman et al. (2020)
Palmitoleoyl [CH_3_-(CH_2_)_5_-CH = CH-(CH_2_)_7_-CO-, *cis*]	NF	D	D	NF	Brady and Clardy (2000)
					Brady and Clardy (2005)
Heptadecanoyl (Margaroyl) [CH_3_-(CH_2_)_15_-CO-]	NF	D	NF	NF	Brady et al. (2004)
Stearoyl [CH_3_-(CH_2_)_16_-CO-]	D,Q	D,Q	D,Q	NF	Tan et al. (2010)
					Tortoriello et al. (2013)
					Leishman et al. (2016)
					Qi et al. (2017)
Petroselinoyl [CH_3_-(CH_2_)_10_-CH = CH-(CH_2_)_4_-CO-, *cis*]	NF	NF	NF	NF	
Oleoyl [CH_3_-(CH_2_)_7_-CH = CH-(CH_2_)_7_-CO-, *cis*]	D,Q	D,Q	D,Q	D	Tan et al. (2010)
					Tortoriello et al. (2013)
					Leishman et al. (2016)
					Kim et al. (2020)
					Zhou et al. (2020)
Elaidoyl [CH_3_-(CH_2_)_7_-CH = CH-(CH_2_)_7_-CO-, *trans*]	NF	NF	NF	NF	
*cis*-Vaccenoyl [CH_3_-(CH_2_)_5_-CH = CH-(CH_2_)_9_-CO-, *cis*]	NF	D	NF	NF	Brady and Clardy (2000)
Vaccenoyl [CH_3_-(CH_2_)_5_-CH = CH-(CH_2_)_9_-CO-, *trans*]	NF	NF	NF	NF	
Linoleoyl [CH_3_-(CH_2_)_4_-CH = CH-CH_2_-CH = CH-(CH_2_)_7_-CO-, all *cis*]	D,Q	D,Q	D,Q	NF	Tortoriello et al. (2013)
					Leishman et al. (2016)
					Long et al. (2016)
*α*-Linolenoyl [CH_3_-CH_2_-(CH = CH-CH_2_)_3_-(CH_2_)_6_-CO-, all *cis*]	NF	D,Q	NF	NF	Liu et al. (2017)
					Berman et al. (2020)
Arachidoyl [CH_3_-(CH_2_)_18_-CO-]	D,Q	NF	NF	NF	Long et al. (2016)
Arachidonoyl [CH_3_-(CH_2_)_4_-(CH = CH-CH_2_)_4_-(CH_2_)_2_-CO-, all *cis*]	D,Q	D,Q	D,Q	D	Tan et al. (2010)
					Leishman et al. (2016)
					Long et al. (2016)
					Borras et al. (2017)
					Berman et al. (2020)
Eicosapentaenoyl [CH_3_-CH_2_-(CH = CH-CH_2_)_5_-(CH_2_)_2_-CO-, all *cis*]	D,Q	NF	NF	NF	Long et al. (2016)
Behenoyl [CH_3_-(CH_2_)_20_-CO-]	NF	NF	NF	NF	
Erucoyl [CH_3_-(CH_2_)_7_-CH = CH-(CH_2_)_11_-CO-, *cis*]	NF	NF	NF	NF	
Docosahexaenoyl [CH_3_-CH_2_-(CH = CH-CH_2_)_6_-CH_2_-CO-, all *cis*]	D,Q	D,Q	D	D	Tan et al. (2010)
					Leishman et al. (2016)
					Long et al. (2016)
Lignoceroyl [CH_3_-(CH_2_)_22_-CO-]	NF	NF	NF	NF	
Nervonoyl [CH_3_-(CH_2_)_7_-CH = CH-(CH_2_)_13_-CO-, *cis*]	NF	NF	NF	NF	

a D, detected, Q = quantified, and NF, nothing found.

Clardy, Brady, and co-workers have found that soil microorganisms may produce many different NA-ArAAs (Brady and Clardy, 2000; Brady et al., 2004; Brady and Clardy, 2005; Clardy and Brady, 2007). The NA-ArAAs included in Table 1 from their work are only those that have been fully identified and characterized. However, these researchers have reported that other NA-ArAAs are produced by soil microorganism, which were not included in Table 1. These were NA-ArAAs that possess a long-chain, monounsaturated acyl group, but the position and stereochemistry of the double bond was not delineated. *N*-Acyl-L-tyrosines with acyl groups of C8:1, C9:1, and C17:1 were generated by environmental *N*-acyl amino acid synthases expressed in *E*. *coli* (Brady et al., 2004).

An examination of the data in Table 1 reveals apparent gaps in our knowledge of the NA-ArAAs from living systems. One key issue is the stereochemistry of the aromatic amino acid. It is not always clear in the work cited in Table 1 that the aromatic amino acid was definitively identified as the l-isomer. A few *N*-acyl-D-aromatic amino acids have been identified (Table 2) and the D-aromatic amino acids are found in man (Table 3). The most is known about the biologically generated *N*-acyl-tyrosines relative to the other NA-ArAAs; there are 27 different *N*-acyl groups attached to Tyr with 13–16 different *N*-acyl groups for His, Trp, and Phe. The identification of a relatively large cohort of *N*-acyl-tyrosines is not simply a consequence of the relatively high cellular concentration of L-Tyr because, at least in human blood, all the aromatic amino acids are approximately the same concentration (20–150 μM, see Wishart et al., 2018). There is a wealth of data on the *N*-acetyl aromatic amino acids, limited to no data for acyl chains of 3–15 carbon atoms in length, and then considerable data for longer-chain acyl groups of 16–22 carbon atoms in length. We did not include acyl-chains longer than 24 carbon atoms in our search because very-long chain fatty acids represent a low percentage of the total fatty acids found in living systems (generally less than 2%), are of limited aqueous solubility, are not well studied (Rezanka and Votruba, 2002; Řezanka and Sigler, 2009). Much of the data for identification of the NA-ArAAs with acyl-chains containing 6–15 carbon atoms comes from the work on the expression of environmental DNA in *E*. *coli* (Brady and Clardy, 2000; Brady et al., 2004; Brady and Clardy, 2005; Clardy and Brady, 2007), suggesting that medium-chain *N*-acyl aromatic amino acids have important cellular and extracellular functions for soil microorganisms. Straight-chain acyl groups predominate the NA-ArAAs listed in Table 1. For branched-chain acyl groups, we found information for only *N*-isovaleroyl- Phe, Tyr, Trp, and His and *N*-(11-methyldodecanoyl)-Phe. Branched chain fatty acids typically represent a small percentage of total cellular fatty acids, <4% (Nicolaides, 1974); thus, the limited data for branched-chain NA-ArAAs may reflect the overall abundance of this class of acyl chains in a living system. The apparent gaps in our knowledge about the NA-ArAAs discussed here may reflect differences in the respective abundance of the fatty acids and the aromatic amino acids in the cell or may be a consequence of search bias or simply that these have not yet been observed. Perhaps, a targeted search for *N*-propionylated or *N*-myristoleoylated aromatic amino acids will demonstrate that these NA-ArAAs are produced by living systems.

**TABLE 2:** *N*-Acyl-D-Aromatic Amino Acids Identified from Living Systems

d-Amino Acids^a^					
*N*-Acyl Group	Phe	Tyr	Trp	His	References
Acetyl [CH_3_-CO-]	D	NF	D	NF	Kaur et al. (2013)
					Gauthier et al. (2018)
					Chahel et al. (2019)
					Mengdi et al. (2020)
					Pinto et al. (2020)
					Rahaman et al. (2021)
Malonyl [HOOC-CH_2_-CO-]	D	NF	D	NF	Sakagami et al. (1995)
					Farag et al. (2016)
Gymnastatin N^b^	NF	D	NF	NF	Phoon et al. (2004)

a D, Detected and NF, nothing found.

b Gymnastatin N = *N*-(2*E*,4*E*,6*R*)-4,6-dimethyl-2, 4-dodecadienoyl-d-tyrosine.

**TABLE 3:** Concentrations of the D-Aromatic Amino Acids in human bodily fluids^a^.

Amino Acid	CSF	Plasma	Urine
d-Phenylalanine	340–2,500 nM	100–300 nM (50–70 nM)^b^	40–250 nM
d-Tyrosine	0–10 nM	0–120 nM (190–470 nM)^b^	30–120 nM
D-Tryptophan	2–5 nM	0–30 nM (80–110 nM)^b^	20–50 nM
d-Histidine	BLD^c^	BLD^c^ (270–630 nM)^b^	50–520 nM

a Data from Visser et al. (2011).

b The values in the parentheses are the rat plasma concentrations (Xing et al., 2016).

c BLD, below the level of detection.

## Biosynthesis

Living systems produce a diversity of NA-ArAAs (Table 1). One set of questions regarding the NA-ArAAs is: 1) how are they made, 2) how are they degraded, 3) how are they metabolized to other metabolites, and 4) how are they transported? There are reports describing an enzyme-catalyzed condensation between an amino acid and a fatty acid to generate a lipo-amino acid, which does not require the addition of ATP or coenzyme A (CoA) (Bachur and Udenfriend, 1966; Katayama et al., 1999; Lait et al., 2003; Koreishi et al., 2006; Long et al., 2016). Catalysis of the direct condensation reaction is often attributed to hydrolases like fatty acid amide hydrolase (FAAH) (Sugiura et al., 2002; Ueda, 2002; Hu et al., 2009). There is no question that hydrolases will catalyze lipo-amino acid formation *in vitro*. However, the significance of this chemistry *in vivo* is unclear because this reverse hydrolase reaction generally requires high concentrations of the substrates and pH values >8. Comparison of the kinetic constants for synthesis vs. hydrolysis reveals that the hydrolase reaction is generally favored by a factor of 10^2^–10^3^ (Katayama et al., 1999; Long et al., 2016). FAAH knock out mice exhibit higher levels of anandamide and other fatty acid amides than wild type mice (Cravatt et al., 2001; Saghatelian et al., 2004; Hu et al., 2009; Han et al., 2013) providing strong evidence that, *in vivo*, the major role of FAAH is in fatty acid amide hydrolysis and not synthesis.

Recent work on the mammalian enzyme, peptidase M20 domain 1 (PM201D1), suggests that this enzyme may catalyze direct condensation of fatty acids and L-Phe to form the long-chain *N*-acylphenylalanines in the serum under physiologically relevant conditions of pH and substrate concentrations (Long et al., 2016; Kim et al., 2020). The binding of PM201D1 to serum lipoprotein particles stimulates its synthesis activity. In addition, the lipo-amino acids bind to serum albumin, which serves to protect the lipo-amino acids from degradation and provides a thermodynamic driving force for synthesis by decreasing the serum concentration of the “free” lipo-amino acid.

One conventional route for the synthesis of NA-ArAAs and the other lipo-amino acids is the nucleophilic attack of the *α*-amino group of the amino acid at the activated carboxyl group of the fatty acid. Biologically, the carboxyl group is activated by formation of an acyl phosphate, an adenylate, or a thioester (like an acyl-CoA). There is evidence that acyl phosphates are substrates for the formation of *N*-acyl amino acids in bacteria (Katz et al., 1953). ATP-grasp enzymes catalyze the ATP-dependent formation of an acyl phosphate, which is then employed for the generation of an amide bond (Iyer et al., 2009; Fawaz et al., 2011). A number of ATP-grasp enzymes that utilize an amino acid as a substrate has been identified, but, to date, there has not been an ATP-grasp enzyme identified for the biosynthesis of an NA-ArAA. Of particular note is the ATP-grasp enzyme, carnosine synthase, which catalyzes the formation of *β*-alanyl-phosphate and ADP from ATP and *β*-alanine. Subsequent attack on the *β*-alanyl-phosphate by histidine yields carnosine and, presumably, inorganic phosphate (Drozak et al., 2010).

The ATP-dependent formation of an activated acyl adenylate is another approach utilized biologically for carboxylate activation (Hara et al., 2018). Fatty acyl adenylates are critical in the formation of acyl-CoA thioesters (Dieckmann et al., 1995). Interception of a fatty acyl adenylate by an aromatic amino acid could yield a NA-ArAA. However, there is no known enzyme-catalyzed transformation that directly involves a fatty acyl adenylate in the formation of an NA-ArAA. NBAD (*β*-alanyldopamine) synthase catalyzes a related reaction in the biosynthesis of *β*-alanylhistamine (carcinine) and NBAD with *β*-alanyl-adenylate as an intermediate (Richardt et al., 2003).

While acyl phosphates and acyl adenylates could have a role in NA-ArAA biosynthesis, acyl thioesters do have a role in NA-ArAA biosynthesis. The *N*-acetylated versions of all the aromatic amino acids are known (Table 1) and transferases that catalyze the acetyl-CoA-dependent formation of *N*-acetyl-Gly (Schachter and Taggart, 1954), Glu (Sonoda and Tatibana, 1983), Asp (Madhavarao et al., 2003), His (Yamada et al., 1995), and Phe (Krishna et al., 1971) are known. There are glycine *N*-acyltransferases that utilize long-chain acyl-CoA thioesters and glycine as substrates yielding the long-chain *N*-acylglycines (Waluk et al., 2010; Jeffries et al., 2016). A variation of this chemistry is the H_2_O_2_-dependent formation of *N*-arachidonoylglycine from arachidonoyl-CoA and glycine in a reaction catalyzed by cytochrome c (McCue et al., 2008). However, acyltransferases responsible for the biosynthesis of *N*-acetyl-Tyr, *N*-acetyl-Trp or the longer-chain NA-ArAAs have not been described. The family GCN5-related *N*-acyltransferases (GNAT) consists of over 10,000 members from all forms of life and most these GNATs have not been characterized. Amongst this group of uncharacterized GNATs, there may be a transferase that will catalyze the acyl-CoA-dependent production of the NA-ArAAs. A GNAT-related enzyme that catalyzes the formation of *N*-acetyl-D- and *N*-succinoyl-D-Phe, D-Tyr, or D-Trp from the aromatic d-amino acid and acetyl-CoA or succinoyl-CoA has been described (Sakai et al., 2006). The proposed final step in the biosynthesis of the gymnastatin N, a fungal cytotoxic metabolite, is the reaction between d-tyrosine and (2*E*,4*E*,6*R*)-4,6-dimethyl-2,4-dodecadienoyl-CoA, as catalyzed by an acyltransferase (Tong et al., 2021). We have identified *N*-arylalkylamine *N*-acyltransferases (AANATs) that accept long-chain acyl-CoA thioesters and arylalkylamines that are related to the aromatic amino acids, like histamine, phenethylamine, tyramine, and tryptamine, but these enzymes will not utilize the aromatic amino acids as substrates (Dempsey et al., 2014; Dempsey et al., 2015; Battistini et al., 2019).

Another acyl thioester involved in NA-ArAA biosynthesis are acyl groups attached to the thiol group of the 4′phosphopantetheine prosthetic group of an acyl carrier protein (ACP); chemistry related to non-ribosomal peptide bond synthesis. The novel *N*-acyl amino acid synthases described by Brady, Clardy, and co-workers utilize long-chain *S*-acyl-ACPs as substrates for the formation of NA-ArAAs (Brady et al., 2002; Brady et al., 2004; Craig and Brady, 2011). The acyl group donors for the formation of the *S*-acyl-ACPs are either the aminoacyl adenylates, the acyl-CoA thioesters, or an acyl-lipoate (Van Wagoner and Clardy, 2006; Craig and Brady, 2011; Paiva et al., 2018).

**FIGURE 2:**
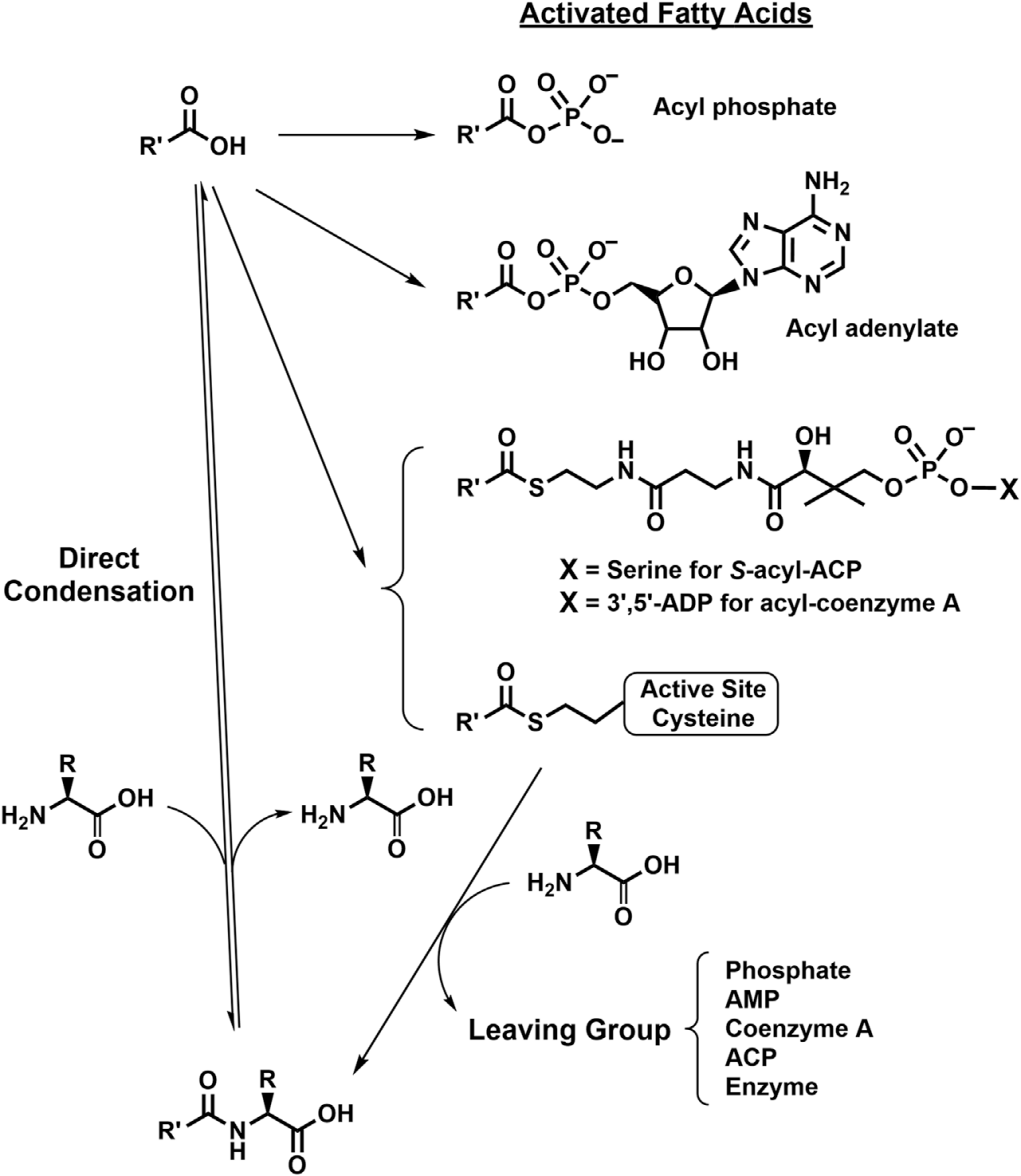
Putative Biosynthetic Routes for the NA-ArAAs. Details for the biosynthesis of the activated fatty acids were omitted because some of the reactions have not been conclusively identified. However, all the activation reactions would likely be ATP-dependent, either directly or indirectly. The R-group represents the side-groups for the aromatic amino acids shown in Figure 1 and R′-CO- represents the acyl groups from Table 1.

Acyl thioesters as intermediates in the cellular production of NA-ArAAs could also occur via an acylated active site Cys. Acylated Cys residues have been identified as catalytically important intermediates for a number of enzymes, but, in each case, the acyl group is transferred from an acyl-CoA to the active site Cys (Palmer et al., 1991; Born and Blanchard, 1999; Wang et al., 2005). Perhaps, there is a CoA- or ATP-independent method to form an active site *S*-acyl-Cys, which would account for the reports of the energy independent direct conjugation of fatty acids to amines. The possible reactions for the biosynthesis of NA-ArAAs are summarized in Figure 2.

Because of their importance in chemical and biochemical processes, amide synthesis is at the heart of many synthetic endeavors. The most obvious method of their synthesis, the direct condensation of a carboxylic acid and amine is energetically unfavorable. The chemical amide synthesis often requires a two-step sequence involving activated carboxylic acid ester, which then undergo aminolysis (Valeur and Bradley, 2009). We have not found an example for the use an activated fatty acid ester in the biosynthesis of an NA-ArAA *in vivo*.

## Degradation

In this review, we have separated degradation from metabolism. Degradation is the hydrolysis of the NA-ArAAs to the corresponding fatty acid and aromatic amino acid. Metabolism (discussed below) is the modification of the fatty acyl and/or the amino acid moiety of the NA-ArAAs to form other metabolites. A balance between biosynthesis and degradation is important in maintaining the cellular concentration of the NA-ArAAs (Di Marzo, 2006; Biringer, 2021) and hydrolase may contribute to the “feed-back” of acyl groups into the biosynthesis of the fatty acid amides (Bradshaw et al., 2009b). Hydrolytic enzymes like fatty acid amide hydrolase (FAAH), *N*-acylethanolamine hydrolyzing acid amidase (NAAA), carnosinase, aminoacylase (amidohydrolase), acylase, and/or deacetylases could catalyze the hydrolytic degradation of the NA-ArAAs. There is evidence that specific NA-ArAAs are substrates for acylases (Nagai, 1961; Endo, 1978; Koreishi et al., 2006) and amidohydrolases (Goldstein, 1976; Denton, 2006; Sakai et al., 2006; Koreishi et al., 2009). However, there is little to no evidence that most of the NA-ArAAs are substrates for any well characterized hydrolase. The amidohydrolase superfamily, >36,000 members, and deacetylase superfamily, >10,000 members, are large and most of these enzymes are uncharacterized (Akiva et al., 2014). Saghatelian et al. (2004) demonstrated that long-chain *N*-acyltaurines are FAAH substrates *in vivo* despite *in vitro* data obtained using purified enzyme showing that the long-chain *N*-acyltaurines are poor FAAH substrates exhibiting relatively low k_cat_/K_M_ values. Saghatelian et al. (2004) found that long-chain *N*-acyltaurines accumulated in the brain and spinal cord of FAAH^−/−^ mice despite low k_cat_/K_M_ values for the long-chain *N*-acyltaurines obtained using purified, recombinant rat FAAH. While species differences between the FAAH sources might account for the inconsistencies between the *in vivo* and *in vitro* data (comparing FAAH −/− mice to purified rat FAAH), Saghatelian et al. (2004) suggest that these discrepancies result from differences between competing biosynthetic and degradative pathways *in vivo*. Note that the pathways for the biosynthesis and degradation of the *N*-acyltaurines, like those for the NA-ArAAs, are not completely understood. None-the-less, the work of Saghatelian et al. (2004) indicates that *in vitro* activity data may not always accurately predict *in vivo* activity. Despite the lack of explicit data, the NA-ArAAs are likely subjected to enzymatic hydrolysis *in vivo*.

## Other Metabolic Pathways

**FIGURE 3:**
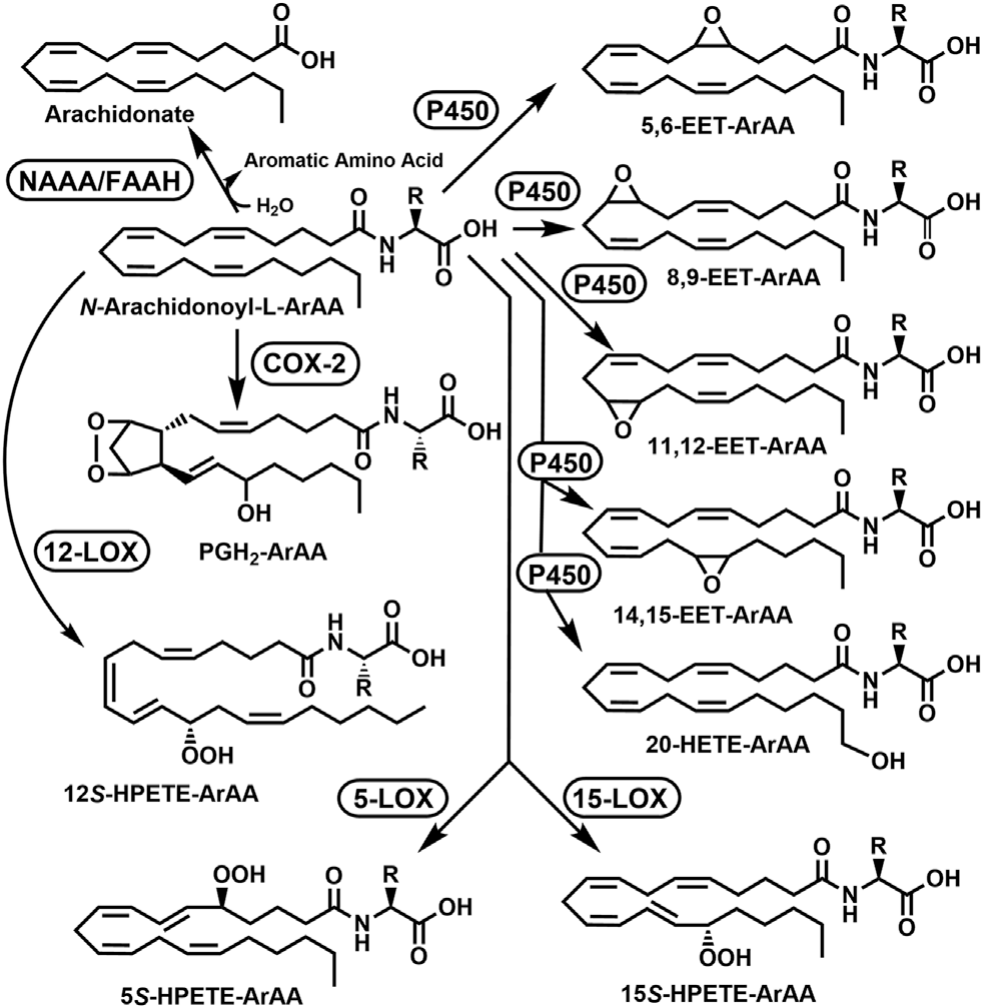
Putative Acyl Group Oxidation and Hydrolytic Degradation of the N-Arachidonoyl Aromatic Amino Acids (ArAAs). The possible metabolites are 5,6-EET-ArAA, *N*-5,6-epoxyeicosatrienoyl-ArAA; 8,9-EET-ArAA, *N*-8,9-epoxyeicosatrienoyl-ArAA; 11,12-EET-ArAA, *N*-11,12-epoxyeicosatrienoyl-ArAA; 14,15-EET-ArAA, *N*-14,15-epoxyeicosatrienoyl-ArAA; 20-HETE-ArAA, *N*-20-hydroxyeicosatetraenoyl-ArAA; 5*S*-HPETE-ArAA, *N*-(5*S*-hydroperoxy)-eicosatetraenoyl-ArAA; 12*S*-HPETE-ArAA, *N*-(12*S*-hydroperoxy)-eicosatetraenoyl-ArAA; 15*S*-HPETE-ArAA, *N*-(15*S*-hydroperoxy)-eicosatetraenoyl-ArAA; and PGH_2_-EA, prostaglandin E_2_-ArAA. The enzymes would be COX-2, cyclooxygenase-2; FAAH, fatty acid amide hydrolase; 5-LOX, 5-lipoxygenase; 12-LOX, 12-lipoxygenase, 15-LOX, 15-lipoxygenase, NAAA, *N*-acylethanolamine hydrolyzing acid amidase; and P450, cytochrome P450. The R-group represents the side-groups for the aromatic amino acids shown in Figure 1. This figure is adapted from the modifications of anandamide described in Rouzer and Marnett (2011) and Biringer (2021).

Reactions to modify the NA-ArAAs are likely to occur at the acyl group and/or the amino acid group. Enzyme catalyzed oxidation/hydroxylation of the acyl moiety of *N*-palmitoylglycine (Haines et al., 2001), *N*-linolenoyl-L-glutamine (Yoshinaga et al., 2005), *N*-arachidonoyltaurine (Turman et al., 2008), *N*-arachidonoylglycine (Prusakiewicz et al., 2002; Prusakiewicz et al., 2007), and anandamide (Rouzer and Marnett, 2011; Biringer, 2021) have been reported. This chemistry has been well described for anandamide with the characterization of the metabolites generated by treatment with cyclooxygenase-2 (COX-2), different lipooxygenases (LOXs), different cytochrome P450s, and FAAH. The anandamide-derived products resulting from COX-2 and LOX catalysis are substrates for additional enzymatic modification leading to >12 anandamide derived metabolites in mammals (Rouzer and Marnett, 2011; Biringer, 2021). To date, there are no studies showing the acyl group of NA-ArAAs has been modified as detailed in this paragraph for other members of the fatty acid amide family. The modifications of arachidonoyl moiety of anandamide, *N*-arachidonoyltaurine, and *N*-arachidonoylglycine serve as a basis for putative modifications of the *N*-arachidonoyl aromatic amino acids (Figure 3).

The conversion of the aromatic amino acids to other metabolites is known, examples include hydroxylation of L-Phe to L-Tyr, conversion of L-Tyr to l-DOPA, epinephrine, and other neurotransmitters, and conversion of L-Trp to serotonin, melatonin, niacin, and kynurenine (Parthasarathy et al., 2018; Liu et al., 2020a).

**FIGURE 4:**
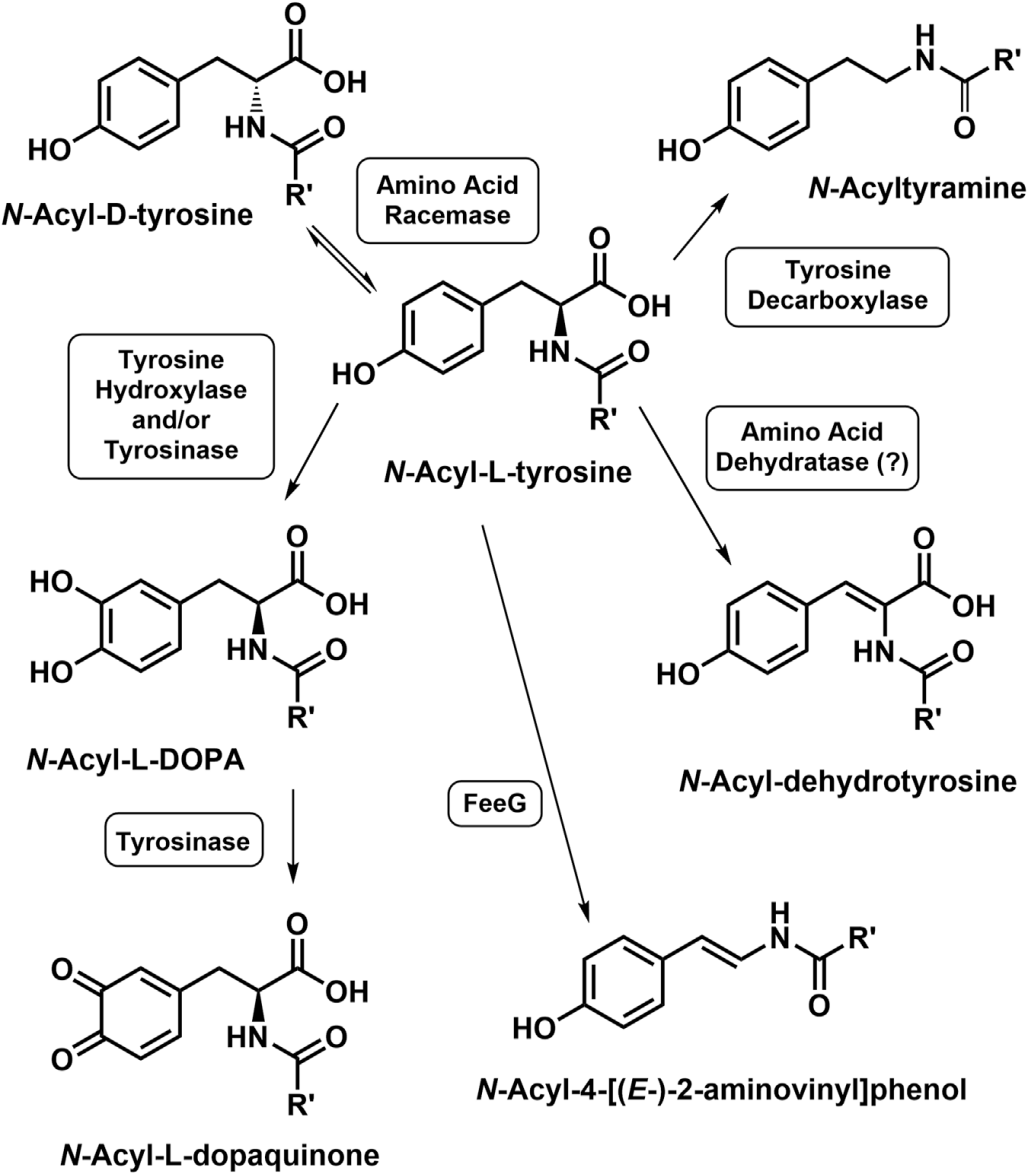
Modifications of the Tyrosyl Moiety of the N-Acyl-L-Tyrosines. An enzyme catalyzing the conversion of *N*-acyl-L-tyrosine to the corresponding *N*-acyl-dehydrotyrosine is unknown, suggested to be an *N*-acyl amino acid dehydratase by Sandargo et al. (2020).

*N*-Acetyl-tyrosine is a substrate for tyrosinase leading to *N*-acetyl-l-DOPA and/or *N*-acetyl-dopaquinone (Kahn and Ben-Shalom, 1998). Decarboxylation of *N*-arachidonoyl-L-tyrosine to *N*-arachidonoyl-l-tyramine was reported using rat tissue homogenates *in vitro* (Akimov et al., 2007). *N*-Acyl-dehydrotyrosines result from the oxidation of *N*-acyl-L-tyrosines, which are biosynthetic intermediates in the formation of 1) the thalassotalates and thalassotalamides in the marine bacterium *Thalassotalea* (Deering et al., 2016) and 2) stieleriancine D in marine plantomycete, *Stieleria neptunia* (Sandargo et al., 2020). Oxidative decarboxylation of the *N*-acyl-L-tyrosines yields the *N*-acyl-4-[(*E*-)-2-aminovinyl] phenols, a reaction catalyzed by the enzyme FeeG from soil bacteria (Brady et al., 2002; Rachid et al., 2010). Amino acid racemases that accept both *N*-acyl-L- and D-NA-ArAAs have been reported (Sakai et al., 2006; De Cesare and Campopiano, 2021). The reactions for the modification of the tyrosyl moiety of the *N*-acyl-L-tyrosine are shown in Figure 4. To the best of our knowledge, no other work has been carried out to determine if the NA-ArAAs are converted to the corresponding *N*-acyl metabolites via the known reactions of aromatic amino acid metabolism. In fact, one function of the NA-ArAAs could be as biosynthetic intermediates in the production of other cellular metabolites.

## Transport

Transport is an issue of considerable debate with the fatty acid amide fields. The fatty acid amides and, in particular, the NA-ArAAs, are of limited aqueous solubility. For example, the critical micelle concentrations for the sodium salts of *N*-stearoyl-L-Phe, Tyr, Trp, and His range from 2.0–2.3 mM (Sivasamy et al., 2001) and that for the sodium salt of *N*-lauroyl-L-Phe is 0.8 mM (Ghosh and Dey, 2015). Within the fatty acid amide family, the most work concerning transport has been carried out for anandamide. There were suggestions that an anadamide transporter did not exist and that anandamide moves across membranes by passive diffusion (Ortega-Gutiérrez et al., 2004; Glaser et al., 2005). However, transport proteins for anandamide have been reported, including members of the fatty acid binding protein (FABP) family (Kaczocha et al., 2009) and a catalytically-deficient FAAH-like protein (Fu et al., 2011). The possibility of anandamide-specific inhibitor remains unclear.

Formation of the *N*-acetyl and *N*-isovaleroyl conjugates of the aromatic amino acids are reactions of the phase II component of the mammalian xenobiotic detoxification system (Grant, 1991). Phase III excretion of these short-chain NA-ArAAs is either by passive diffusion or is transporter-mediated (Döring and Petzinger, 2014). Transport of the longer-chain NA-ArAAs shown in Table 1 has not been explicitly investigated. Passive diffusion out of the cell or between organelles is one possible mode of transport for such hydrophobic molecules. Alternatively, the NA-ArAAs could be substrates for either unknown or known transporters. Perhaps, a member of solute carrier (SLC) superfamily, specially a monocarboxylate transporter (MCT) or an L-type amino acid transporter (LAT), or a member of the organic anion transporter (OAT) family (Pizzagalli et al., 2021) will mediate the movement of the NA-ArAAs across the cell membrane. Long et al. (2016) provide evidence that NA-ArAAs bind to members of the SLC25 family of mitochondrial solute carriers. MCT10 is responsible for aromatic amino acid transport in mammals (Halestrap, 2013) and, thus, might transport the NA-ArAAs. NA-ArAA transport is a topic that requires additional research.

## Bioactivity

As mentioned above, the NA-ArAAs could serve as biosynthetic intermediates in both mammalian and non-mammalian orgranisms. In mammals, the short-chain NA-ArAAs are likely involved in the detoxification of high cellular levels of short-chain organic acids and/or aromatic amino acids because of a metabolic defect. One example is the presence of *N*-acetyl-L-phenylalanine in the blood and urine of people suffering from phenylketonuria (Goldstein, 1963; Bonte et al., 2019).

The cellular functions for most of longer-chain NA-ArAAs, the lipo-amino acids, and the other members of the fatty acid amide family are largely unknown. Data for the two best studied fatty acid amides, anandamide and oleamide, indicate that these are cell signaling molecules in mammals. Anandamide binds to the two cannabinoid receptors, CB_1_ and CB_2_, and may exert its cellular effects by binding to other receptors (Devane et al., 1992; Maccarrone et al., 2015; Pacher et al., 2020). Oleamide regulates the sleep/wake cycle, blocks gap junction communication (Guan et al., 1997), and exhibits other neuromodulatory activities (Fedorova et al., 2001). The cellular functions of oleamide result from its binding to CB_1_, (Leggett et al., 2004), specific members of serotonin receptor family, 5-HT_1A_, 5-HT_2A_, 5-HT_2C_, and 5-HT_7_ (Huidobro-Toro and Harris, 1996; Boger et al., 1998; Thomas et al., 1999), and, possibly other receptors, as well (Karwad et al., 2019).

Cellular functions in mammals have been suggested for a few of the lipo-amino acids (Hanuš et al., 2014; Burstein, 2018; Battista et al., 2019; Prakash and Kamlekar, 2021). Long-chain *N*-acylated L-phenylalanines, *N*-palmitoyl, *N*-oleoyl, and *N*-linoleoyl, are uncouplers of the uncoupling protein 1 (UPC1)-independent respiration in mitochondria and may contribute to the regulation of glucose homoeostasis (Long et al., 2016). Other cellular functions attributed to NA-ArAAs in mammals include the antiproliferative effects of *N*-palmitoyl-L-tyrosine against cultured human breast cancer cells (Burstein and Salmonsen, 2008) and the neuroprotective effects of *N*-stearoyl-L-tyrosine and *N*-linoleoyl-L-tyrosine (Zhang et al., 2009; Liu et al., 2020b). The cellular activities of the NA-ArAAs and other lipo-amino acids may involve their binding to one of the following receptors: GPR18, GPR55, GPR92, and/or GPR132 (Bradshaw et al., 2009a; Burstein, 2018). The cellular presence of the NA-ArAAs may contribute to the “entourage effect.” The “entourage effect” refers to endocannabinoids or cannabinoid-related molecules that exhibit no direct interaction with a protein/enzyme involved in fatty acid amide metabolism, but enhance the cellular activity of related molecules (Pacher et al., 2020). By serving as alternative substrates for the degradative hydrolases, the NA-ArAAs could foster a higher and sustained concentration of anandamide—the “entourage effect”.

In non-mammalian organisms, the cellular function of the NA-ArAAs, lipo-amino acids, and the other fatty acid amides are not well understood. The NA-ArAAs may function as biosurfactants (Mhaskar et al., 1990; Joondan et al., 2017), which are important in microorganisms for defense, motion, and metabolism (Banat et al., 2014); or as antibacterials (Brady and Clardy, 2000; Brady et al., 2004; Brady and Clardy, 2005; Clardy and Brady, 2007; Lee et al., 2019), in the battle between soil bacteria for limited resources.

The cannabinoid receptors are absent in insects (McPartland et al., 2001), yet the NA-ArAAs and other lipo-amino acids are produced by insects (Tortoriello et al., 2013; Jeffries et al., 2014; Anderson et al., 2018). Thus, the NA-ArAAs may not be cell signaling molecules in insects or the insect signaling pathways are different from those found in mammals. Truitt et al., 2004 report that volicitin, a lipo-amino acid produced in insects, binds tightly (*K*_d_ = 1.3 nM) to a protein found in a plant, *Zea mays*. A specific NA-ArAA might be produced in one organism to elicit a response in a different organism, an issue that could dramatically complicate defining function for that NA-ArAA. Cellular function(s) for the NA-ArAAs is a largely unanswered and challenging question. MALDI mass spectrometry imaging (Genangeli et al., 2019) and the use of a NA-ArAA-based photoaffinity probe are two approaches that might help solve the question of cellular function (Long et al., 2016; Merkler and Leahy, 2018).

## N-Acyl Aromatic Amino Acids as Artifacts?

One nagging question for anyone working on the fatty acid amides or the lipo-amino acids is the following: “Aren’t these molecules simply artifacts of breaking open the cells to look for them?” This question or one similar is posed during seminars and in reviews of manuscripts and grant applications. This question is not new. Wren (1960) posed this question over 60 years ago. This question is not unfair. The process of destroying cellular integrity will expose free amines to activated carboxylates leading to amide formation. The relatively low levels of the lipo-amino acids found and the variability in the levels fosters a concern about artifacts. All enzymes must catalyze the conversion of products to substrates. Thus, hydrolases must catalyze “reverse hydrolysis,” the direct conjugation of an amine to a free carboxylate. In their analysis of PM201D1, Long et al. (2016) determined that the amount of *N*-oleoyl-L-phenylalanine generated was consistent with the K_eq_ value and the cellular concentrations of L-Phe and oleate.

The data shows that the identification and characterization of NA-ArAAs, lipo-amino acids, and free fatty acids from living organisms cannot be dismissed as artifacts, exactly as concluded by Wren (1960). Enzymes that catalyze the synthesis of these molecules *in vitro* have been discovered and knock-out experiments lead to the accumulation of expected precursors (Cravatt et al., 2001; Hu et al., 2009; Han et al., 2013). In fact, the *N*-acyltaurines were first discovered in FAAH^−/−^ mice (Saghatelian et al., 2004). The cellular levels of anandamide are regulated by a balance between biosynthesis and degradation (Di Marzo, 2006; Biringer, 2021). Similar data on the cellular levels of the NA-ArAAs do not exist, but provide evidence that a related molecule is not an artifact of the isolation methodology. Also, the identification of a relatively large cohort of *N*-acyl-tyrosines (Table 1) despite approximately the same concentration of all the aromatic amino acids, at least in human blood, is not coincidental but suggests to a more specific catalytic origin. The possibility of artifacts regarding the identification of the NA-ArAAs from living system is a concern, but a concern that can be eliminated with careful and proper controls.

## Discussion

Provided here is a thorough review of our knowledge of the NA-ArAAs, covering those that have been identified from a living system, how these lipoamino acids could be made, degraded, and metabolized within a cell, and how these molecules could be transported out between organelles and out of the cell. Lastly, we discuss the data which shows that NA-ArAAs are not merely an artifact of disrupting cells.

Our review points towards a number of areas concerning the NA-ArAAs that require further research. We have highlighted the gaps in our knowledge of about the biologically-occuring NA-ArAAs included in Table 1 and have proposed that these gaps may have resulted from a search bias. Future searches may show that many of the NA-ArAAs “missing” from Table 1, including those possessing a D-aromatic amino acid, may exist in a living system.

Clearly, significant questions remain about the biosynthesis, degradation, metabolic conversion, transport, and bioactivity of the NA-ArAAs. Given the importance of anandamide and oleamide in mammals, the structurally related NA-ArAAs are likely of significance in living systems. A more complete understanding of the NA-ArAAs will likely lead to a better understanding of human health, identify new targets to treat human disease and control the pests that spread disease and damage our crops, and enhance their uses as food additives, drug delivery agents, and in the cosmetic and pharmaceutical industries (Chhikara et al., 2011; Kong et al., 2011; Taresco et al., 2016; Bernal et al., 2018; Tripathy et al., 2018).
